# Memory for pictures of sexual assault: Sensitive maintenance of ambiguous stimuli

**DOI:** 10.1371/journal.pone.0236873

**Published:** 2020-07-29

**Authors:** Jan Hendrik Peters, Michael Hock

**Affiliations:** Department of Psychology, University of Bamberg, Bamberg, Germany; University of Padova, ITALY

## Abstract

Individual differences in dispositional coping might influence how ambiguous situations involving interactions of men and women are interpreted and remembered. Specifically, we hypothesized that women with a sensitive coping style actively maintain ambiguously threatening stimuli in their memory, showing so-called sensitive maintenance. As a prerequisite to investigate this hypothesis, two surveys (Studies 1 and 2; *N* = 151 and *N* = 252) were conducted to answer the questions whether fear of sexual assault is of relevance for young women in Germany and whether ambiguous (rather than only unambiguously threatening) situations are experienced to a significant extent. After confirming this for our target population, our main hypothesis was tested in Study 3 (*N* = 192) by combining tasks assessing the appraisal and the forgetting of nonthreatening, threatening, and ambiguous pictures showing interactions of men and women, and by varying the cognitive load during the retention interval. Whereas fear of rape predicted the appraisal of pictures, coping dispositions predicted forgetting of ambiguously and unambiguously threatening pictures in the hypothesized way. Results are discussed from the perspective of adaptivity and functionality of memory.

## Introduction

Rape and, more generally, sexual assault are severe crimes and it has been stated that rape is the crime that women fear most (e.g., [1, 2]). Despite extensive research about the consequences of assault (for an overview, see [3]), few studies have examined the fear of rape and assault in *non-victimized* women (for exceptions, see [4–7]). Due to fear of assault, women often take precautionary measures to reduce their risk. For example, they avoid certain places and means of transport or refrain from wearing clothes that might be interpreted as provoking [4, 8, 9]. Thus, it seems worthwhile to more thoroughly investigate the fear of sexual assault among non-victimized women.

To partly fill this research gap, we investigated (a) the associations of dispositional coping (vigilance and cognitive avoidance) with women’s perception of the threat of sexual assault and (b) the role coping dispositions play in how well women remember threatening and threat-related ambiguous interactions between men and women. We hypothesized that persons with a vigilant compared to a cognitively avoidant coping style would exhibit a better long-term memory for threatening and, especially, for ambiguous interactions. The cognitive process supposed to underlie this memory difference is *sensitive maintenance*, which refers to the spontaneous rehearsal and refreshing of threat-related stimuli. Sensitive maintenance is an active process executed primarily by vigilant persons, but not by persons with other coping styles [10, 11].

We define *sexual assault* as an intentional sexual act without the person’s consent that is committed by physical force, coercion, abuse of authority or when the person was not able to give valid consent (e.g., due to alcohol or drugs). This includes, for example, rape, attempted rape, touching of the person’s genitals, touching of unclothed breast, tongue kissing, masturbation in front of the person (cf. [12]). Although it is difficult to draw a clear line between sexual assault and sexual harassment, for the use of the present research we define *sexual harassment* as unwanted and offensive, sexually toned acts like, for example, unwelcome touching of clothed body parts, hugging and non-intimate kissing, sexually suggestive comments and jokes, offensive gestures, whistling (cf. [13]).

### Coping dispositions and memory for threatening information

Persons who are exposed to the same threat-related situation can perceive it in different ways and, consequently, experience different levels of anxiety. This variation is partly due to coping dispositions and personality traits associated with individual differences in coping. Moreover, in accordance with their coping disposition, persons differ in their memory for threat-related situations (e.g., [14]).

Two fundamental coping dispositions are vigilance and cognitive avoidance. *Vigilance* describes the orientation toward threat and the intensified processing of threat-related information. *Cognitive avoidance* refers to averting attention from threat and inhibiting the further processing of such information. Vigilance and cognitive avoidance are related to the personality dimensions *intolerance of uncertainty* and *intolerance of emotional arousal*, respectively (e.g., [15, 16]). Persons with a high intolerance of uncertainty experience discomfort if it is uncertain how a threatening situation will develop. To reduce uncertainty, they manifest vigilance by gathering as much information as possible, even if the information is threatening. By contrast, persons with a high intolerance of emotional arousal find it difficult to stand the physical and emotional arousal that accompanies the perception of threatening information. Consequently, they refrain from processing threat-related information, thus exhibiting cognitive avoidance. Following the traditional terminology in personality-oriented coping research [17], persons who (due to their high intolerance of uncertainty) consistently manifest vigilance in threatening situations are called “sensitizers,” whereas persons who (due to their high intolerance of emotional arousal) consistently manifest cognitive avoidance are called “repressors.”

Coping dispositions are associated with the memory for threatening and ambiguous information. The most prominent memory phenomenon is that repressors—in the long term—remember fewer self-threatening events than sensitizers do (e.g., [18–20]). Previous explanations for these memory differences have focused on encoding or retrieval mechanisms [21–25] (for overviews, see [14, 26]). However, coping-related memory differences critically depend on the length of the retention interval. This becomes evident in studies that contrasted memory for threatening material in immediate and delayed tests: Compared to sensitizers, repressors showed poor memory for threat on delayed tests but relatively good memory in immediate tests [11, 26–28]. Consequently, accounts that rely solely on encoding or retrieval mechanisms cannot explain the observed time course of forgetting in repressors and sensitizers.

Peters et al. [10] hypothesized that this specific time course of forgetting is primarily caused by sensitizers’ active maintenance processes, such as repeated refreshing and rehearsal of threatening stimuli during the retention interval. To test this hypothesis, the authors conducted two experiments in which they presented participants with threatening and nonthreatening pictures or words, respectively. This presentation was followed by immediate and delayed recognition tests. Between these tests, participants were assigned to a high or to a low cognitive load task in order to prevent (high load) or allow (low load) sensitive maintenance. Under low cognitive load, sensitizers forgot far less of the threatening stimuli than repressors did. Under high cognitive load, however, compared to repressors, sensitizers did not exhibit less forgetting of threatening stimuli. For nonthreatening stimuli, there were no memory differences between sensitizers and repressors. These results support the hypothesis that sensitizers spontaneously engage in cognitive processes of maintaining threatening information, whereas repressors do not (see also [11]).

The present paper aims at replicating the process of sensitive maintenance for women’s retention of assault-related stimuli and at extending it to ambiguous stimuli. As we will discuss, assault-related stimuli are especially suitable for investigating whether ambiguous stimuli are subject to sensitive maintenance or whether this process is restricted to unambiguously threatening material.

### The ambiguity in situations of fear of sexual assault

Fear of sexual assault (including rape) in women is widespread (e.g., [29]) and was even described as “an ever-present, all-pervasive threat by most women” [5]. Although this fear is based on real threats (e.g., [1, 29]), it can be questioned whether the objective likelihood to be victimized is appropriately estimated. At least, potential perpetrators are often located incorrectly (e.g., [30]): Situations feared by women frequently represent the prototypical stranger rape (e.g., being attacked at night in a park by a stranger); however, about 80 to 90% of all rape incidents in the EU and the USA are acquaintance or partner rapes [4, 9, 29, 31]. For the EU [29], representative data support an overestimation of the perceived risk to be victimized by a stranger compared to the perceived risk of assault by a known person. Presumably, the wrong localization of potential offenders in those countries is partly due to mass media reports and movies giving a distorted picture of reality [32].

It can be assumed that many women experience more often ambiguous than unambiguously dangerous situations [33]. To illustrate what is meant by an ambiguous situation we cite what Gordon and Riger [34] have described as a prototypical situation for experiencing fear of assault for women living in the USA: A woman is walking down an unlit street and observes a man walking toward her. Her heart starts pounding. She thinks about whether he might have bad intentions, how she could get help or protect herself, and so on. As the man approaches, he walks past her and continues down the street. We call this an *ambiguous* situation due to the absence of perceivable signs of any bad intention of the man (such signs would turn the situation into an unambiguous threat situation). It has been assumed that the perceived risk of such ambiguous situations often exceeds the objective risk [2, 32].

It seems likely that experiencing ambiguous situations contributes substantially to women’s fear of sexual assault, especially if those situations are experienced more often than unambiguous threat situations. We hypothesize that sensitizers remember ambiguous situations especially well because they fear unanticipated situations for which they are not prepared. To cope with this fear, they search information about upcoming events and consider in advance all possible incidents they might encounter in a particular situation.

### Overview of the present research

More than 30 years ago, Warr [2] reported that women (under the age of 35) in the USA fear rape more than any other crime. To confirm that fear of sexual assault is also of relevance for our target sample of Study 3 (female students in Germany), we conducted two surveys, one as a paper-pencil version with female students of psychology (Study 1) and one on the internet with a demographically more heterogenous sample of German speaking women (Study 2). In these studies, we also assessed how often women experience situations of ambiguous and unambiguous threat and to which sources they ascribe their fear of assault. It should be noted that Study 1 and 2 were not designed to gain a representative picture of the prevalence of fear of sexual assault or exact numbers of ambiguous and unambiguous situations experienced by women. The aims were merely (a) to test whether sexual assault is threatening for our target sample so that corresponding pictures, which were used as stimuli in Study 3, would work to elicit fear and (b) to test our assumption that ambiguously threatening situations are remembered to a significant extent.

After confirming that fear of sexual assault is highly prevalent in our target sample, and ambiguous situations are reported as even more common than unambiguously threatening situations, we designed Study 3 to answer our main research questions. First, we examined how coping styles affect the interpretation of ambiguous pictures that depict interactions of men and women. We expected that sensitizers, compared to other persons (especially repressors), would tend to interpret ambiguous pictures as more threatening. Second, we wanted to test whether sensitive maintenance applies to ambiguous or only to unambiguously threatening stimuli. Therefore, after the presentation and rating of pictures, participants underwent a recognition test for half of the pictures. Then, participants completed a task that imposed either high or low cognitive load on their working memory. Finally, the remaining half of the pictures was tested for recognition. This procedure resembled the two experiments in Peters et al. [10] and allowed us to calculate a forgetting score. We expected that sensitizers under low cognitive load would show little forgetting of ambiguous and of threatening pictures. In contrast, when under high cognitive load, sensitizers were expected to exhibit increased forgetting of these stimuli. There were two controls: (a) Repressors should not show this pattern (i.e., they should forget as much threatening and ambiguous material under low as under high cognitive load) and (b) no coping-related differences in forgetting should be found for nonthreatening material.

Moreover, we assumed that forgetting is mostly influenced by dispositional coping and not by anxiety. To test this, we included measures of trait anxiety, fear of rape, rape-avoidance behavior, and appraisal of pictures in our model.

## Study 1 and 2

Study 1 and 2 were conducted as a prerequisite to Study 3 to answer the following questions: (a) Is fear of sexual assault widespread and of high relevance among young (educated) women in Germany (i.e., in a sample similar to the one to be collected for Study 3)? (b) Is the assumption that ambiguous situations are more common than clearly threatening situations justified for this population? As a non-essential but interesting side aspect we wanted to know: (c) To what sources do these women attribute their fear of sexual assault? Additionally, we assessed the lifetime prevalence of rape in the two samples.

Study 2 was a methodically close replication of Study 1 but used the internet to collect data. The main purpose of Study 2 was to ensure that the findings of Study 1 hold not only for a very specific sample of female psychology students but also for a more heterogenous sample of German women that was, however, similar to the sample expected for Study 3. For a better comparison of both studies, we report them together.

### Method

#### Participants

Participation was voluntary. Participants were informed that the data collection was completely anonymous and that they could terminate the investigation at any time. Informed consent was obtained in written form at the beginning of the studies. All studies reported in this article were reviewed and approved by Ethics Board of the University of Bamberg.

Participants of Study 1 were 151 female undergraduate university students (age: *M* = 22.5 years, *SD* = 5.4 years) studying psychology. Participants were recruited by notices on bulletin boards and announcements in lectures and received course credit for participation.

Participants of Study 2 were recruited by announcements on different internet forums and social networking platforms like Facebook; the announcements were placed by student assistants in groups they had some kind of connection to (e.g., sport clubs, choir, co-workers) but that were unrelated to the topic of violence against women, and it was asked to share these posts to other people and spread the request to participate. Data collection was terminated when 280 participants had completed the online questionnaire. Seventeen participants with more than 2% missing responses were deleted. Moreover, 11 participants showing clearly suspicious response patterns (e.g., same answer on all items) or obviously inconsistent responses (e.g., perceiving it as less severe to be murdered than to be robbed) were excluded from further analysis. The 252 remaining participants were women aged between 18 and 61 years (*M* = 25.6, *SD* = 7.6). About 72% were students; further occupations varied widely, including child care worker, teachers, physicians, craftswomen, homemakers, and businesswomen.

#### Material

The questionnaire contained three sections corresponding to the research questions stated above. First, following the study by Warr [2], we described several crimes and negative life events listed in Table 1. For each of these events, participants rated their fear (1 = *very little* to 10 = *very much*), the likelihood of the event during their lifetime (in percent), and the severity of the event (1 = *not severe* to 10 = *very severe*). Second, participants stated how often during a year they experience ambiguous and unambiguous situations of sexual threat. Third, we listed different possible sources of the fear of sexual assault (see S1 Table). Participants indicated how strongly they agree that their fear stems from these sources (1 = *completely disagree* to 7 = *completely agree*). Finally, participants indicated whether they had ever experienced rape, and if so, by which perpetrator (stranger, acquaintance, friend, relative, partner).

**Table 1:** Fear, likelihood of experiencing (Lh), and severity of specific crimes and negative events in Study 1 (*N* = 151) and Study 2 (*N* = 252).

Item	Study 1						Study 2					
	Fear		Lh (%)		Severity		Fear		Lh (%)		Severity	
	*M*	*SD*	*M*	*SD*	*M*	*SD*	*M*	*SD*	*M*	*SD*	*M*	*SD*
Car accident with serious injuries due to someone else’s carelessness	6.3	2.7	28.6	26.5	8.5	1.6	6.1	2.5	28.5	25.0	8.6	1.5
Raped by stranger	5.7	2.9	7.7	12.5	9.5	1.2	5.9	3.1	14.5	21.2	9.7	0.7
Rape (perpetrator not specified)	5.6	2.8	8.4	14.0	9.5	1.2	6.0	3.0	17.5	25.5	9.8	0.6
Burglarized while at home	5.6	2.9	10.3	15.3	8.4	1.7	5.1	3.0	10.7	15.5	8.1	1.8
Burglarized while not at home	4.7	2.4	19.6	20.0	6.9	1.9	4.7	2.6	21.2	21.9	6.6	2.0
Robbed	4.4	2.3	23.2	19.2	5.6	2.0	4.4	2.2	23.2	22.9	6.0	2.0
Murdered	4.2	3.1	1.6	5.0	9.5	1.7	4.3	3.3	4.3	8.4	9.8	0.8
Computer notebook destroyed by carelessness of someone else	3.7	2.5	26.6	24.2	4.7	2.3	3.3	2.4	26.2	25.1	4.1	2.2
Obscene phone call	2.8	2.0	23.6	25.2	4.1	2.2	3.2	2.3	25.6	29.4	4.2	2.2
Raped by partner	2.5	2.7	3.2	11.6	9.5	1.5	3.0	3.1	5.9	16.9	9.7	1.0

Fear and severity were assessed on scales from 1 to 10, with higher values expressing greater fear/severity.

#### Procedure

In Study 1, participants were invited in groups of 3 to 10 persons into a lecture room where they filled in the questionnaire. To demonstratively ensure anonymity, participants dropped their questionnaires into a closed box.

For Study 2, participants found a link leading to the online survey implemented by SoSci Survey (www.soscisurvey.de) in the announcements of the study. Participants could answer the survey from their homes or any other place they chose and worked through the online questionnaire by themselves. No data were collected that could have jeopardized anonymity.

### Results and discussion

Statistics describing fear, likelihood, and severity of crimes and events are presented in Table 1. The events most feared are car accidents leading to severe injuries (Study 1 and 2: *M*s = 6.3 and 6.1), stranger rape (*M*s = 5.7 and 5.9) and rape by a not specified perpetrator (*M*s = 5.6 and 6.0). Being raped by a partner (*M*s = 2.5 and 3.0) was feared significantly less than being raped by a stranger (*M*s = 5.7 and 5.9), Study 1: *t*(150) = 12.66, *p* < .001, *d* = 1.15; Study 2: *t*(251) = 15.71, *p* < .001, *d* = 0.96. By contrast, being raped by a partner (Study 1 and 2: *M*s = 9.5 and 9.7) was judged as *severe* as being raped by a stranger (*M*s = 9.5 and 9.7). Moreover, the *likelihood* of being raped by a stranger (Study 1 and 2: 7.7% and 14.5%) was considered more than twice as high as being raped by a partner (Study 1 and 2: 3.2% and 5.9%), Study 1: *t*(150) = 3.45, *p* < .001; Study 2: *t*(251) = 5.64, *p* < .001. This explains why stranger rape is feared more than partner rape. The comparison of data for stranger rape with rape without specified perpetrator indicates that women generally associate rape with stranger rape. Remarkably, the perceived likelihoods for stranger and partner rape are at odds with the actual rape experiences: Our participants reported a lifetime prevalence of rape of 7.9% and 14.7% in Study 1 and 2, respectively. Partner rape had been experienced by 6.0% and 8.3%, stranger rape by 0.7% and 3.2% (some participants reported rape by acquaintances, friends or relatives). This is in accordance with previous studies demonstrating that partner rape is more prevalent than stranger rape (e.g., [31]).

Having a car accident with serious injuries (*M*s = 8.5 and 8.6) was not judged as severe as being raped by a stranger (*M*s = 9.5 and 9.7), Study 1: *t*(149) = 8.09, *p* < .001, *d* = 0.68; Study 2: *t*(250) = 11.06, *p* < .001, *d* = 0.97. However, the car accident was considered to be far more likely (*M*s = 28.6% and 28.5%) than stranger rape (*M*s = 7.7% and 14.5%), Study 1: *t*(150) = 10.54, *p* < .001; Study 2: *t*(251) = 8.53, *p* < .001. This explains why serious car accidents were most feared by our participants.

In conclusion, the perceived severity of being raped is extremely high, regardless of the perpetrator. The observation that stranger rape (but not partner rape) was highly feared in the present samples can partly be explained by the fact that our participants estimated stranger rape to be more than twice as likely as partner rape. As a car accident does not constitute a crime, the statement by Warr [2] that rape is the crime women fear most could be confirmed with our samples—at least for the crimes included in our questionnaires.

Our second research question dealt with the remembered prevalence of ambiguous and unambiguous situations of sexual threat. Because the distributions of answers were extremely positively skewed (skewness between 3.9 and 6.0), we report medians in addition to means. The average frequencies of remembered unambiguous situations per year were *M* = 0.83, *Mdn* = 0.00, *SD* = 2.33, in Study 1, and *M* = 1.27, *Mdn* = 0.25, *SD* = 2.41, in Study 2. As is evident from the medians, the majority of women reported experiencing less than one situation per year. Regarding remembered ambiguous situations, corresponding results were *M* = 13.40, *Mdn* = 4.00, *SD* = 29.11, in Study 1, and *M* = 16.29, *Mdn* = 5.00, *SD* = 41.15, in Study 2. Consequently, ambiguous situations of sexual threat were reported much more often than unambiguous situations. This difference is highly significant, regardless of the applied measure of central tendency, *p*s < .001.

Retrospective reporting over a period of 12 month can be compromised by various distortions during the processes of memory consolidation and recall; for emotional events as in the case of ambiguous and unambiguous situations of sexual threat several cognitive and neurocognitive mechanisms improving as well as impairing retrieval have been discussed (cf. [35, 36]). Therefore, one should be very cautious in interpreting the absolute numbers of reported ambiguous and unambiguous threat situations. However, in view of the fact that the reported number of ambiguous compared to unambiguous situations is so much higher (depending on the study and the measure of central tendency at least 12 times higher), it seems unlikely that memory distortions could affect ambiguous and unambiguous situations so markedly different that the true relation would be reversed (i.e., that unambiguous threat situations are experienced more often than ambiguous situations). The observed difference in retrospective reporting of ambiguous and unambiguous threat situations at least supports the notion that for many women ambiguous situations are relevant enough to be remembered. Moreover, there was considerable variability regarding the number of ambiguous and unambiguous situations women reported. This variability may partly be due to real differences in the number of incidents women are exposed to, however, individual differences in the interpretation and/or memory of such situations as explored in Study 3 may also contribute to it.

Our third research question concerned to which sources women attribute their fear of sexual assault. In the self-reports of our participants, personal experiences (Study 1 and 2: *M*s = 2.1 and 3.0) and experiences told by personally known women (*M*s = 2.5 and 3.5) contributed relatively little to the fear of assault. Mass media reports like news on television (*M*s = 5.6 and 4.7) and newspaper reports (*M*s = 5.1 and 4.3) contributed the most to anxiety. This confirms the hypothesis that fear of sexual assault is not primarily based on personal experiences but on cognitive schemata conveyed through media, at least in a sample of relative young women in Germany. More detailed data regarding this research question can be found in the (S1 Table).

In sum, the results of the surveys support our hypothesis that fear of sexual assault is a salient fear for our target sample. Importantly, ambiguous threat situations are reported to be experienced far more frequently than unambiguous threat situations The participants report to believe that their fear of sexual assault is fostered primarily by mass media and only to a small amount by personal experiences.

## Study 3

### Method

#### Experimental design

We applied a 2 (intervening task: high vs. low cognitive load) × 2 (recognition delay: immediate vs. delayed) × 3 (threat: threatening vs. ambiguous vs. nonthreatening stimuli) design. Only the first variation was between subjects: Participants were either prevented from rehearsing pictures during the retention interval due to high cognitive load or had cognitive resources available for maintenance processes due to low cognitive load. The second variation allowed the calculation of the forgetting score: The amount of forgetting during the retention interval was indexed by the recognition performance on the immediate test minus the recognition performance on the delayed test. The third variation served to test whether sensitive maintenance applies to ambiguous as well as to unambiguous threat material. Non-threatening pictures served as a control condition under which no effects of sensitive maintenance are expected.

#### Participants

Of the 195 women participating in the study, three were excluded from the analysis because their memory performance on the immediate test was at chance level. The remaining 192 participants were between 18 and 46 years old (*M* = 23.3, *SD* = 4.0 years). Most of them (*n* = 178) were students from various fields (most prevalent fields were undergraduate psychology: *n* = 43, teaching for primary and secondary schools: *n* = 39, education/social work: *n* = 26, sociology/political sciences: *n* = 22, economics: *n* = 19, languages: *n* = 12, natural sciences: *n* = 5). Nine participants reported as their main occupation to be in employment and five participants went to school (equivalent to senior high school).

Based on results of previous studies [10, 11], we expected low to moderate effect sizes for the interesting associations involving sensitive maintenance (i.e., *ρ* about.20). With an alpha error of.05 and a power of.80 this leads to an *N* of 153 participants for directional research hypotheses. To further increase statistical power, we collected data of about 40 additional participants. Since our data analysis included structural equation modeling, we additionally used a newer method for power analysis that specifically addresses path models, the so-called inverse square root method proposed by Kock and Hadaya [37]. According to Kock and Hadaya, this method leads to conservative estimates. Interestingly, for a standardized path coefficient of *β* = .20, the inverse square root method yields again a sample size of *N* = 153. In addition, we conducted a small simulation study using the R package paramtest [38] for addressing the power for an interaction term present in our main model. The simulation, which can be found in the S1 Text, shows that our sample size is sufficient to reveal the presence of an interaction of low to moderate size (i.e., at least 4% explained variance; see [39]).

Participants were recruited through postings on the campus and via email distribution lists that contained e-mail adresses of individuals who had previously indicated their general interest in participating in research of the psychology department. We declared that the study investigated how women perceive the threat of rape and that victimized women should not participate because some of the pictures may have strong emotional effects on them. As a consequence, the sample consisted mainly of non-victimized women. The experiment lasted approximately 2.5 hours for which participants received 20 Euros. Participants were randomly assigned to the cognitive-load groups, resulting in 94 participants in the high and 98 participants in the low cognitive load group.

#### Procedure

Experiments were run individually by female experimenters. Participants were informed in written form before they came into the laboratory that the study was about women’s fear of sexual assault, how they coped with this fear, and that they would view pictures depicting violence. (However, to prevent the anticipation of recognition tests, we avoided any clues to the fact that the experiment was concerned with memory.) At the beginning of the experiment, this information was repeated verbally by the experimenter and participants were told that they could stop the experiment at any time without any disadvantages for them. This information was also handed out in written form to the participants who confirmed verbally that they had understood the information and that all additional questions had been answered. After the experiment, participants received contact information of a trained psychologist that could be contacted at no charge if they had the impression that the experiment had triggered anything they wanted to talk about. This study was also reviewed and approved by Ethics Board of the University of Bamberg.

After providing informed consent, participants completed questionnaires that assessed *coping dispositions* and *trait anxiety*. Then, the *picture appraisal* and the *memory task* were administered on a computer (19-inch screen, 1,240 × 1,028 resolution). The computer tasks were followed by a final questionnaire that included scales on *fear of rape* and *rape-avoidance behavior*.

#### Questionnaires

The relevant questionnaires are described below. Additionally, participants responded to filler questionnaires during a 5-min delay before the first recognition test and during a 9-min recreational break between the two parts of the cognitive load task.

To assess *vigilance and cognitive avoidance*, participants completed the physical threat version of the Mainz Coping Inventory (MCI; [40]). This inventory consists of four physically threatening situations (e.g., approaching a group of suspicious-looking people while walking alone at night in a side-alley). Each scenario is followed by 10 scenario-specific items that assess either vigilance (5 items, e.g., “I observe their behavior closely”) or cognitive avoidance (5 items, e.g., “I say to myself that these people are certainly innocuous”). Participants indicate whether they generally react this way in the given situation. In the present sample, vigilance and cognitive avoidance yielded acceptable internal consistencies (*α*s = .76 and .74, respectively). Because vigilance and cognitive avoidance were substantially correlated (*r* = -.52, *p* < .001), we calculated a vigilance minus cognitive avoidance difference score, in this way obtaining a bipolar scale for assessing coping styles. High positive scores on this scale indicate consistent vigilance (i.e., sensitization), whereas high negative scores indicate consistent avoidance (i.e., repression). This vigilance-avoidance score yielded a Cronbach’s *α* of.83.

For assessing *general trait anxiety*, participants completed the State-Trait Anxiety-Depression Inventory (STADI; [41]), which allows the separation of anxiety and depression [42]. The trait-anxiety scale had an *α* of.89.

To assess the presence of *fear of rape* in the participants’ daily lives, we used a German 11-item version of the Fear of Rape Scale [33], based on a translation by Krahé [6]. Example items are “I often think how likely it is to become the victim of rape” and “I believe that many men can become sexually dangerous.” Cronbach’s *α* was.85.

The *rape-avoidance scale* aims to assess everyday behavior that women exhibit to avoid exposure to potentially dangerous situations. The 25 items were adapted from a questionnaire established by Krahé [5, 6]. Typical items are “When I am out at night, I try to walk in the company of other women” and “I try to wear clothes in which I can defend myself.” Cronbach’s *α* was.88.

A table with descriptive statistics of the questionnaires is given in S1 File. Means and standard deviations of the variables were similar to previous studies.

#### Appraisal task

Participants viewed a series of pictures, one at a time, depicting one female and one or several male actors. Their task was to rate how threatening they perceive the situation shown in the picture. To heighten self-referent processing of the material, participants were instructed to mentally place themselves in the position of the woman in the picture.

In each trial, a centered fixation cross shown for 400 ms was followed by the centered picture (400 × 400 pixels) for 300 ms. Participants immediately rated whether they appraised the situation to be threatening, ambiguous, or non-threatening by pressing one of three corresponding keys on a response pad. We instructed participants to answer quickly but to avoid careless mistakes. Trials were separated for 2 s by a blank screen. A total of 180 trials were presented (60 trials each contained threatening, ambiguous, and non-threatening pictures). To avoid serial position effects, each person received the trials in one of two fixed randomized orders. The experimental trials were preceded by 30 practice trials.

The photographs of interactions were obtained from the International Affective Picture System [43] and by Internet searches. To ensure that the memory task was difficult enough to index forgetting during an interval of 40 min, test stimuli and distractors were matched closely with regard to pictorial content and colors in sets of 3 (level of threat) × 2 (set 1 vs. set 2) pictures. The division into set 1 and 2 served to balance test stimuli and distractors between participants in the recognition tests.

In a pilot study, 14 female students rated over 900 pictures on *threat to oneself* (1 = *not at all* to 9 = *very threatening*), *ambiguity* (1 = *not at all* to 9 = *very ambiguous*), and *valence* (1 = *very positive* to 9 = *very negative*). Based on these ratings, we chose the 360 best-fitting pictures. For cross-validation, these pictures were rated by another 10 women. In this final rating, threatening pictures were appraised as very threatening (*M*_threat_ = 8.2, *SD*_threat_ = 0.9), very negative (*M*_valence_ = 7.9, *SD*_valence_ = 0.8), and only slightly ambiguous (*M*_ambiguity_ = 2.6, *SD*_ambiguity_ = 1.8). Ambiguous pictures had relatively high ratings on ambiguity (*M*_ambiguity_ = 6.0, *SD*_ambiguity_ = 1.7), midpoint ratings on threat (*M*_threat_ = 4.9, *SD*_threat_ = 1.3), and neutral ratings on valence (*M*_valence_ = 5.8, *SD*_valence_ = 1.1). Nonthreatening pictures were rated as not threatening (*M*_threat_ = 1.3, *SD*_threat_ = 1.6), unambiguous (*M*_ambiguity_ = 1.6, *SD*_ambiguity_ = 1.1), and slightly positive (*M*_valence_ = 3.0, *SD*_valence_ = 1.2). Ratings for set 1 and set 2 pictures matched very closely (differences between sets did not exceed 0.25 scale points and were all nonsignificant).

An appraisal score was constructed by coding threat appraisals of pictures as 2, ambiguous appraisal as 1, and nonthreat appraisals as 0, respectively. The resulting score represents the tendency of persons to appraise pictures encountered during the experimental session as threatening. The construction of such an overall score seems justified as the appraisals of threatening, ambiguous, and non-threatening stimuli were highly correlated, and the mean score of the class-specific appraisals had a satisfactory reliability (*α* = .75).

#### Memory task

The memory task consisted of (a) the initial recognition test 1, (b) an intervening task (high vs. low cognitive load), and (c) the delayed recognition test 2. The set of pictures presented to the participant in the picture appraisal task (i.e., the pictures serving as test stimuli in the recognition tests) was split again into two halves, each containing 30 threatening, ambiguous, and non-threatening pictures. The subset that was assigned to the first and second recognition tests was balanced between participants. In each recognition test, old pictures were complemented by an equal number of threatening, ambiguous, and non-threatening “new” pictures serving as distractors.

To diminish recency effects, we inserted a 5-min delay (filled with filler questionnaires) before the first recognition test. The trials of the recognition test were constructed as follows: A single picture was presented in the center of the screen and remained there until participants provided their recognition judgment on a scale from 1 (*certainly presented before = old*) to 6 (*certainly not yet presented = new*). Participants were told to respond without time pressure but not to excessively think about single pictures. The intertrial interval was 500 ms. Trials were presented in a fixed randomized order.

The aim of the high cognitive load condition was to prevent participants from rehearsing and elaborating upon the previously presented pictures. In accordance with the working memory theory of Baddeley [44], we imposed cognitive load on the visuospatial sketch pad using a pictorial test of attention, the Frankfurt Attention Inventory (FAIR; [45]). The FAIR consists of rows of geometric line drawings, called icons, and the participant’s task is to mark all icons of a specific type. Because the icons in the FAIR are highly distinct from the photographs used as memory stimuli, effects of memory interference are minimized. Participants in the high cognitive load condition were instructed to work as quickly and as accurately as possible. To avoid tiring participants too much, they were set under cognitive load two times for 15 min each, separated by a 9-min break, in which a filler questionnaire was completed.

Participants in the low cognitive load condition completed a drawing task that consisted of integrating simple symbols on a sheet of paper into small drawings. They could draw whatever they wished and had plenty of time. This task provided participants with a plausible activity in the context of a psychological study but left sufficient cognitive resources available to rehearse and elaborate upon the previously viewed pictures. After 15 min, participants received the same questionnaire as the high cognitive load group and then continued with a similar drawing task for 15 min. Thus, the procedures of the two cognitive load conditions matched closely. Together with the instruction time, the intervening task lasted 40 min for each group.

After the high/low cognitive load tasks were finished, participants completed a second recognition test. This test was identical to the first recognition test except that participants judged a new set of test stimuli and distractors.

The *recognition score*, indicating the ability to discriminate between test stimuli and distractors, was calculated by subtracting the mean rating of old pictures from the mean rating of new pictures. As the rating scale used ranged 1 (*certainly old*) to 6 (*certainly new*), a recognition score of 5 indicates perfect discrimination, whereas a score of 0 indicates chance discrimination. Our main interest was in the association of coping disposition and forgetting. Therefore, we calculated a *forgetting score* by subtracting the recognition score on the second test from the recognition score on the first test. Thus, higher scores on this variable indicate more forgetting. We examined the general effects of recognition delay (test 1 vs. 2), threat category (threatening vs. ambiguous vs. nonthreatening), and cognitive load during the retention interval (high vs. low) with a three-way ANOVA. This analysis yielded a strong main effect of the recognition delay (test 1: *M* = 2.08, *SD* = 0.56; test 2: *M* = 1.50, *SD* = 0.55), *F*(1, 190) = 584.91, *p* < .001, $\eta_{p}^{2}=.76$, indicating substantial forgetting during the retention interval. Moreover, a main effect of threat category was found, *F*(2, 189) = 64.77, *p* < .001, $\eta_{p}^{2}=.41$: Ambiguous pictures (*M* = 1.96, *SD* = 0.62) were remembered better than threatening (*M* = 1.70, *SD* = 0.55) and nonthreatening (*M* = 1.71, *SD* = 0.57) pictures. The main effect of the cognitive load condition and all other interaction effects were nonsignificant, all *F*s < 1.

### Results

Table 2 presents the correlations among the anxiety and coping measures assessed in the present study. All correlations between the four questionnaire scales were in the expected direction and highly significant (*p* < .01). The highest correlation was found between fear of rape and self-reported rape-avoidance behavior (*r* = .69). The vigilance-avoidance score was strongly associated with trait anxiety (*r* = .50) and fear of rape (*r* = .47). However, the association of vigilance-avoidance and self-reported rape-avoidance behavior was only moderate (*r* = .37). The same was true for the association of trait anxiety and fear of rape (*r* = .37). Trait anxiety was only weakly correlated with rape-avoidance behavior (*r* = .24).

**Table 2:** Correlations of anxiety and coping measures.

Variable	FR	RAB	VIG-AV	Appraisal
Trait anxiety	.37	.24	.50	.21
Fear of rape (FR)		.69	.47	.38
Rape-avoidance behavior (self-report; RAB)			.37	.34
Vigilance-avoidance score (VIG-AV)				.19

*N* = 192. *p* < .01 for |*r*| ≥ .19.

**Fig 1:**
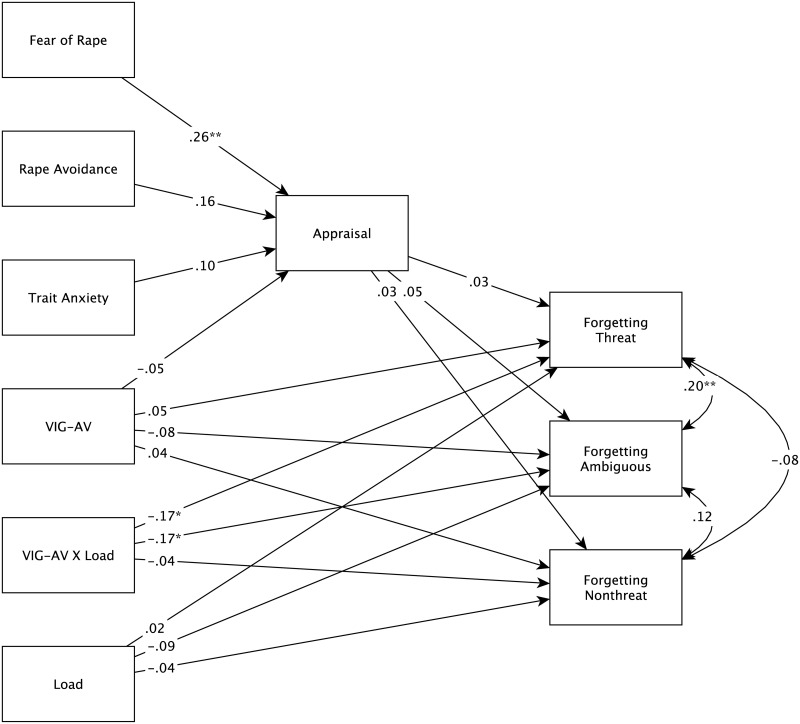
Structural model representing the relations between personality variables, experimental condition, appraisal, and forgetting. Covariances between fixed exogenous variables and residual variances were omitted. LOAD = experimental condition, VIG-AV = vigilance-avoidance score. *^p < .05, **p < .01^ (two-tailed).

Structural equation modeling (SEM) was used to analyze the data. The hypothesis that sensitizers show less forgetting of ambiguous and/or threatening pictures than other people under low load but increased forgetting of these stimuli under high load should lead to an interaction between vigilance-avoidance and the experimental condition. As shown in Fig 1, the model encompassed fear of rape, rape avoidance, anxiety, and the vigilance-avoidance score as purely explanatory variables. To model the hypothesized interaction between vigilance-avoidance and the experimental condition with respect to the forgetting of threatening and ambiguous pictures, an indicator of the condition (coded −1 for high load and + 1 for low load) as well as the vigilance-avoidance by condition product term were included. Appraisals were considered as a potential mediator of the relationships between the personality variables and the forgetting scores. The three forgetting scores were entered as purely dependent variables.

The advantage of a SEM approach over a more traditional approach, in which a series of separate regression analyses and/or ANOVAs are carried out, is fourfold: (a) the fit of the total model can be checked; (b) it is possible to include potential mediators in the analysis; (c) not only hypothesized associations but also assumed (conditional) independencies are tested; (d) non-independence in the criteria is accounted for.

The model was fitted with the *sem* function of the R package Lavaan [46]. We used maximum likelihood estimation with robust standard errors and a Satorra-Bentler scaled test statistic. The fit of the model was good, *χ*^2^ = 11.23, *df* = 11, *p* = .42, RMSEA = .011 (90% confidence interval:.00−.079), SRMR = .027, CFI = .995 [47, 48]. Fig 1 shows the standardized path coefficients.

As indicated by the zero-order correlations, all personality variables were significantly associated with the appraisals of the pictures (Table 2). In the path model, however, only the strongest predictor, fear of rape, remained significant. (For reasons outlined above, we used an aggregated measure of appraisal, which combined ratings of threatening, ambiguous, and nonthreatening pictures. The associations of the personality variables with the appraisals of different types of pictures were very similar. For example, with fear of rape, the correlations were .35, .36, and .29, *p*s < .01. Thus, fear of rape was generally associated with biased threat appraisal of the pictures).

Thus, rape avoidance, anxiety, and vigilance-avoidance do not explain much specific variance in appraisals, over and above fear of rape. The three paths (standardized coefficients, *β*) from the appraisals to the forgetting scores were small and nonsignificant (*p* ≤ .05), indicating that individual differences in appraisals do not act as mediators of the relation between personality and memory variables. Finally, significant interactions between vigilance-avoidance and the load condition on forgetting emerged for both threatening (*β* = −.17, *p* = .014) and ambiguous (*β* = −.17, *p* = .011) pictures, whereas the corresponding interaction for non-threatening pictures was negligible (*β* = −.04, *p* = .54). All remaining regression paths were small. The good fit of the model indicates that, as hypothesized (and in contrast to vigilance-avoidance), fear of rape, rape avoidance, and anxiety do not have substantial associations with the memory variables. To formally test this assumption, we contrasted our model with a model that—in addition to the paths shown in Fig 1—included influences of fear of rape, avoidance, and anxiety on the memory scores. This model failed to show a better fit than our more restrictive model, *χ*^2^ = 9.11, *df* = 9, *p* = .43, indicating that the influences of the anxiety variables on memory were essentially zero.

**Fig 2:**
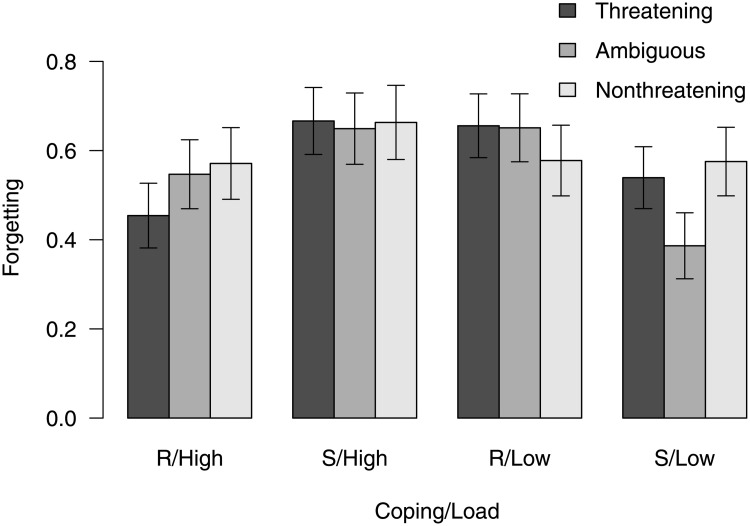
Forgetting scores. Forgetting of threatening, ambiguous, and nonthreatening stimuli under high and low load conditions in repressors (R, predicted values for persons 1 *SD* below the mean of the vigilance-avoidance score) and sensitizers (S, predicted values for persons 1 *SD* above the mean of the vigilance-avoidance score). The lines represent ±1 *SE* around the predicted values.

Fig 2 shows the predicted forgetting scores of sensitizers (persons 1 *SD* above the mean of the vigilance-avoidance score) and repressors (persons 1 *SD* below the mean of the vigilance-avoidance score) under the two load conditions. The predictions were computed from the coefficients of the path model (Fig 1). As evident from Fig 2, under low cognitive load sensitizers showed very low forgetting of ambiguous pictures. In this condition, sensitizers forgot substantially less than repressors did. They also forgot substantially fewer ambiguous pictures than sensitizers under high load did. These results support our hypothesis of sensitive maintenance of ambiguous pictures. The hypothesis that sensitive maintenance occurs only under low cognitive load is corroborated by the fact that under high cognitive load, sensitizers did not forget fewer—but indeed more—ambiguous pictures than repressors. Similarly, albeit less pronouncedly, differences between sensitizers and repressors were found for threatening pictures. Again, under low load, sensitizers showed less forgetting than repressors, whereas under high load they manifested more forgetting.

### Discussion

#### Appraisal

Fear of rape was the best predictor of the threat appraisals of the presented stimuli. Trait anxiety, as a more nonspecific indicator of anxiety, did not contribute substantially to the appraisal ratings. The same was true for vigilance-avoidance. At first glance this is somewhat at odds with previous research that established relations between coping dispositions and the interpretation of ambiguous stimuli (e.g., [26–28]), however, these studies realized longer intervals between stimulus onset and response, turning appraisal into a late—and presumably more strategic—stage in information processing. In the present study, participants provided their picture ratings quickly (within slightly more than one second). Possibly, *early* stages of information processing are more driven by the level of specific anxiety than by more strategic coping processes.

#### Forgetting

As hypothesized, the forgetting of ambiguous pictures under the low cognitive load condition was substantially associated with vigilance-avoidance, with sensitizers manifesting less forgetting than repressors. This effect did not appear under high cognitive load, which is in line with the assumption that sensitive maintenance is an active process that demands cognitive resources [10]. For threatening pictures there was a similar tendency. As shown by testing an extended structural model with direct paths from anxiety to the memory variables, coping dispositions rather than anxiety indicators play the major role in modulating memory processes for threatening and ambiguous pictures.

#### Anxiety, coping, and rape-avoidance behavior

By using general as well as assault-specific measures of anxiety and coping, we are able to address relations between these measures, especially with respect to actions that women might undertake in everyday life to cope with the threat of sexual assault. Trait anxiety and fear of rape were only moderately correlated, which points to the need for the domain-specific assessment of anxiety. As discussed, only the domain-specific anxiety (not general trait anxiety) was a powerful predictor of appraisal.

Vigilance-avoidance and self-reported rape-avoidance behavior were also substantially correlated, with vigilant persons reporting comparatively much behavioral avoidance. Moreover, the association between fear of rape and the reporting of behavioral avoidance was quite close, indicating that women with high fear of rape exhibit a variety of precautionary behavioral strategies to diminish their actual risk of being sexually assaulted.

## General discussion

The present experiment established that sensitive maintenance applies to ambiguous stimuli. We assume that sensitive maintenance is especially strong for ambiguous stimuli if such stimuli are highly relevant for future coping, which should be the case for stimuli related to sexual assault. However, sensitive maintenance is also applied to unambiguously threatening information [10, 11]. Future experiments should test sensitive maintenance with other specific anxieties of high prevalence such as dental fear or test anxiety.

Our findings correspond closely with theories of goal-directed memory (e.g., [49, 50]) which posit that, in particular, incidents that assist the person in coping with present and future threats and challenges are kept retrievable. For the successful behavioral coping with future threats of assault, it is potentially useful to be vigilant and exhibit sensitive maintenance to remember as many ambiguous situations as possible. For sensitizers, this is probably the best strategy because these individuals are able to tolerate the current emotional arousal that accompanies such information processing. For repressors, however, the emotional arousal elicited by the processing of threat-related stimuli would often exceed the level they can tolerate. Consequently, they withdraw attention from such information for the sake of momentary well-being (but probably at the expense of being less prepared for future incidents). These considerations find their equivalent in age-related differences of emotional memory: Older persons, who are likely to experience relatively few new situations with which they must cope, show a poorer memory for threatening stimuli than younger persons do. For younger persons, the benefit of preparing for future challenges is greater, even at the cost of momentary uneasiness (e.g., [49–51]; for associations with coping styles, cf. [52]).

Regarding the interpretation bias found in the appraisals of the pictures, to our knowledge the present study is the first to show that domain-specific anxiety predicts appraisals better than the coping disposition. However, the time dependency of this finding must be examined further: Does the impact of coping dispositions on interpretation biases increase with time [53, 54]? Given the superior predictive power of a specific anxiety measure above a general anxiety measure in the appraisal task, another reasonable approach would be to develop measures of domain-specific cognitive coping.

Moreover, we found in Study 1 and 2 that participants remembered substantially more ambiguous than unambiguous situations. As already mentioned, due to intervening cognitive processes, it is not legitimate to equate the number of retrieved incidences with the number of experienced incidences. On the level of encoding, for example, whether an ambiguous situation is interpreted as threatening or as nonthreatening may partially depend on the fear of assault. In a person with a vigilant coping style, sensitive maintenance will keep threatening situations accessible. Conversely, it is plausible that fear of rape depends on the number of threatening incidents represented in and accessible from memory. Therefore, vigilant persons might get caught up in a vicious circle in which fear of rape and the memory of threatening situations are reciprocally reinforced.

Fear of rape is strongly associated with self-reported rape-avoidance behavior. As we have discussed, rape-avoidance behavior restricts women’s freedom of behavior and their participation in public life (e.g., [34]). This sacrifice pays off even less when one takes into account that most precautionary measures aim at stranger rape, whereas (at least for women in the EU) most rape incidents are acquaintance or partner rapes [29]. In recent years, acquaintance and partner rapes have increasingly become a subject in the public and the mass media. However, Anderson [31] did not find that the perception of the typical rape incident as stranger rape had changed. Similarly, Study 1 and 2 provided no indications of such a change among young women in Germany. Future research with more representative samples should monitor changes in risk perception. However, not perceiving partner rape as a prevalent risk seems to be an essential prerequisite for everyday living in any partnership. These aspects demand thorough investigation.

One limitation of the present studies is the reliance on self-report measures of anxiety and coping. With respect to rape-avoidance behavior, more comprehensive behavioral measures should be developed and applied, especially because the congruency of self-report and behavior in this domain has not yet been established. A further limitation of the present work is that the perceived prevalence of ambiguously and unambiguously threatening situations was assessed retrospectively. A more thorough operationalization could be realized with a diary or an ambulatory assessment method. This could, in combination with retrospective reports, also illuminate how the distortion of memory of real-life events differs depending on individual differences in dispositional coping and other personality traits.

In conclusion, applied research on the fear of rape in non-victimized women can benefit from considering cognitive coping processes and from implementing experimental paradigms that help to uncover these processes. This has rarely been attempted in the past (for exceptions, see [5, 6]). Moreover, the more fundamental research on coping dispositions, encoding, appraisal, interpretation, and memory might profit from examining specific but widespread anxieties such as fear of sexual assault. Stimuli from such domains are often more intense than the typically used generally threatening stimuli. Additionally, our results show that it is important not only to contrast unambiguously threatening with non-threatening stimuli, but also to take into account ambiguous stimuli. After all, ambiguous situations might matter most for future coping.

## Supporting information

S1 Table Sources to which women attribute their fear of rape and sexual violence. (PDF)

S1 Text Simulation: Power curves for an interaction between a binary and a continuous variable. (PDF)

S1 File Zip file containing data for all studies and R script for the analysis of the data of Study 3. (ZIP)
