# Supplementary material for: Memory for pictures of sexual assault: Sensitive maintenance of ambiguous stimuli
Source: PLoS One. 2020 Jul 29;15(7):e0236873. doi: 10.1371/journal.pone.0236873 (PMC7390341; doi:10.1371/journal.pone.0236873)
Supplement: S1 Table — (PDF) [file pone.0236873.s001.pdf]

S1 Table. Sources to which women attribute their fear of rape and sexual violence in Study 1 ($N = 151$) and Study 2 ($N = 252$)

	Study 1		Study 2	
Source	*M*	*SD*	*M*	*SD*
Personal experiences	2.07	1.68	3.02	2.38
Experiences told by personally known women	2.48	1.87	3.54	2.31
Warnings heard in school by teachers	3.11	1.76	2.77	1.88
Warnings by parents	3.72	2.09	3.35	2.02
Discussions on television	4.28	1.93	3.69	2.02
Feature films	4.89	1.92	3.87	1.89
Documentary films	4.96	1.83	4.24	1.88
Reports in newspaper	5.11	1.69	4.29	1.88
News on television	5.65	1.66	4.70	1.90

Note. Answer scale ranged from 1 (*completely disagree*) to 7 (*completely agree*).
