# Supplementary material for: Memory for pictures of sexual assault: Sensitive maintenance of ambiguous stimuli
Source: PLoS One. 2020 Jul 29;15(7):e0236873. doi: 10.1371/journal.pone.0236873 (PMC7390341; doi:10.1371/journal.pone.0236873)
Supplement: S1 Text — (PDF) [file pone.0236873.s002.pdf]

Simulation: Power curves for an interaction between a binary and a continuous variable

Supporting information:
Memory for pictures of sexual assault: Sensitive maintenance of ambiguous stimuli
by Jan H. Peters and Michael Hock

This document contains a small simulation study to obtain power curves for an interaction between a binary and a continuous variable at various effect sizes. Power curves plot the power of a statistical test as a function of the sample size of a study for specific effect sizes and thereby help to decide on the sample size needed for a study. The R code given below runs the simulation and produces the power curves. For running the code, the following packages must be installed and loaded:

```
library(tidyverse)
library(ggplot2)
library(paramtest)
```

We used the R package "paramtest" for running the simulation (Hughes, 2017). This package makes it straightforward to construct power curves for specific models. The setup of the simulation follows the detailed descriptions in the vignette of the package, which is also available here. In essence, three steps are involved to run the simulation. First, a function is defined that runs the computation once with certain parameters. Second, the function is called repeatedly with varying parameter values and the results are stored. Third, the interesting variables in the result are summarized (e.g., by a plot).

The following code performs Step 1 and contains the function that runs the computation. `x1` represents the continuous variable (which is drawn from a standard normal distribution), `x2` represents the binary variable. The latter is coded with `-1` and `1`. This coding implies that the regression coefficient for the interaction (`b3`) is standardized if the continuous variable is drawn from a standard normal distribution. As in the Study 3, the two predictors are uncorrelated by design. The significance level is set to 0.05.

```r
run_regression <- function(simNum, N, b1 = 0, b2 = 0, b3,
    b0 = 0, x1m = 0, x1sd = 1, one.sided = TRUE) {

    x1 <- rnorm(N, 0, 1)
    n1 <- round(N / 2)
    x2 <- c(rep(-1, n1), rep(1, N - n1))
    yvar <- sqrt(1 - b1^2 - b2^2 - b3^2)
    # b3 is standardized when x1 is standard normal and the
    # coding of the binary predictor is -1, +1.
    y <- rnorm(N, b0 + b1*x1 + b2*x2 + b3*x1*x2, yvar)
    model0 <- lm(y ~ x1 + x2) # no interaction
    model <- lm(y ~ x1 * x2)  # interaction
    summ0 <- summary(model0)
    summ <- summary(model)

    # Output from model
    if (one.sided) {
        p.crit <- 0.10
    } else {
        p.crit <- 0.05
    }
    est_x1 <- coef(summ)['x1', 'Estimate']
    p_x1 <- coef(summ)['x1', 'Pr(>|t|)']
    sig_x1 <- p_x1 < p.crit
    est_x2 <- coef(summ)['x2', 'Estimate']
    p_x2 <- coef(summ)['x2', 'Pr(>|t|)']
    sig_x2 <- p_x2 < p.crit
    est_int <- coef(summ)['x1:x2', 'Estimate']
    p_int <- coef(summ)['x1:x2', 'Pr(>|t|)']
    sig_int <- p_int < p.crit
    # f^2 measure of Cohen (1988)
    f2 <- (summ$r.squared- summ0$r.squared) /
        (1 - summ$r.squared)

    return(c(est_x1 = est_x1, p_x1 = p_x1, sig_x1 = sig_x1,
        est_x2 = est_x2, p_x2 = p_x2,
        sig_x2 = sig_x2,
        est_int = est_int, p_int = p_int, sig_int = sig_int,
        f2 = f2,
```

```
        R2 = summ$r.squared,
        yvar = yvar,
        ysd = sd(y)))
}
```

Step 2 runs the function with varying sample and effect sizes, and for one- and two-sided tests. The standardized regression coefficient for the interaction is b3. The sizes of this coefficient are chosen so that they cover low to medium sizes of Cohen's (1988) f^2 effect size measure for regressions (see formula 9.2.2 on page 410): A coefficient of 0.14 corresponds to a f^2 of 0.02 (a small effect size) and a coefficient of 0.36 corresponds to a f^2 of 0.15 (medium effect size). The argument `n.iter` specifies the number of simulations per run.

```
power_res <- grid_search(run_regression,
    params=list(N = seq(50, 300, by = 50),
        b3 = c(0.14, 0.20, 0.30, 0.36),
        one.sided = c(TRUE, FALSE)),
    n.iter = 2000, output='data.frame',
    # The following two settings are machine dependent and
    # should be adapted (or deleted)
    parallel='multicore',
    ncpus=4)
```

```
## Running 96,000 tests...
```

In Step 3, the results of the simulation are processed for plotting and then actually plotted.

```
res <- results(power_res)
res <- rename(res, N = N.test, beta = b3.test,
    Test = one.sided.test) %>%
    mutate(beta = as.character(beta),
        Test =
            if_else(Test == TRUE, "one-sided", "two-sided")) %>%
    group_by(Test, N, beta) %>%
    summarise(
        Power = mean(sig_int),
        m_f2 = mean(f2)
```

```
    )
p <- ggplot(res, aes(N, Power, color = beta)) +
    geom_point() + geom_smooth() +
    scale_x_continuous(n.breaks = 10) +
    scale_y_continuous(n.breaks = 10) +
    geom_hline(yintercept = 0.8) +
    facet_grid(cols = vars(Test))
print(p)
```

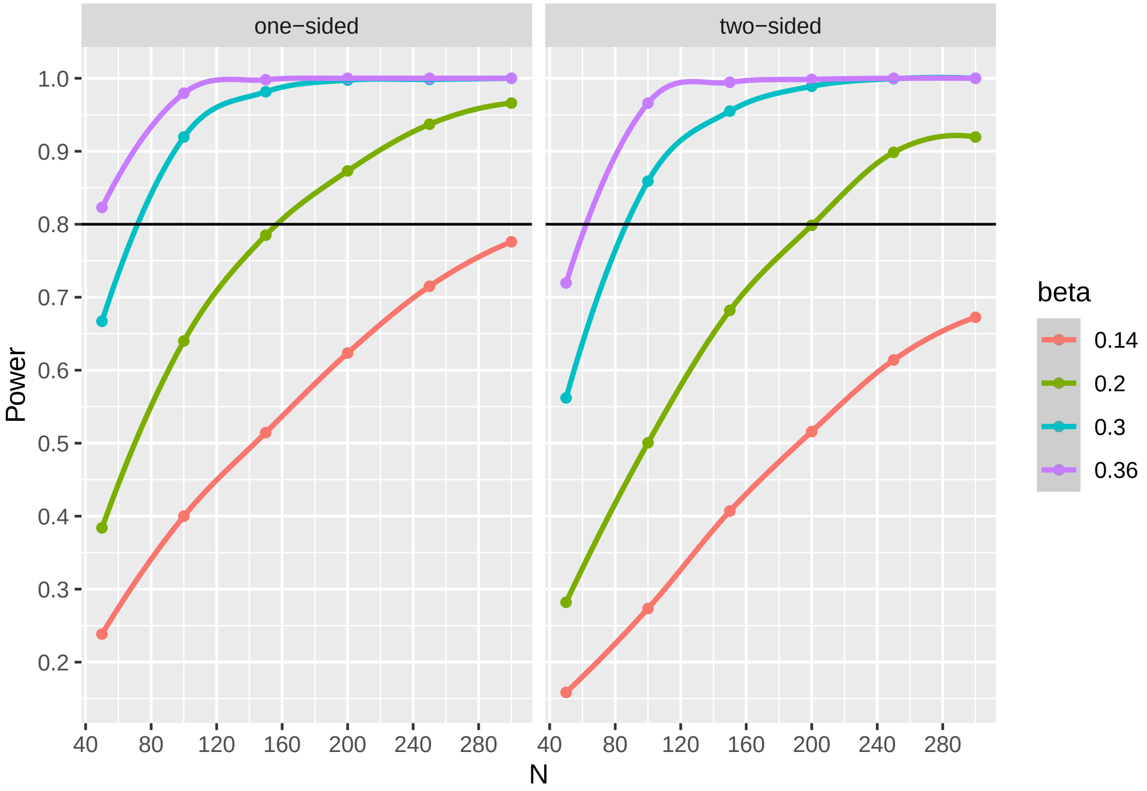

The plot shows that the sample size of Study 3 ($N = 192$) is sufficient to reveal the presence of an interaction of low to moderate size, that is, with a standardized regression coefficient of 0.2 or larger. This coefficient corresponds to an f^2 of 0.042. The conclusion holds irrespective of whether the tests are done one-sided or two-sided. Of course, the one-sided tests are more appropriate in the present context than the two-sided tests because directional hypotheses were formulated.

References

Cohen, J. (1988). *Statistical power analysis for the behavioral sciences* (2nd ed.). Erlbaum.

Hughes, J. (2017). paramtest: Run a function iteratively while varying parameters [R package version 0.1.0]. https://CRAN.R-project.org/package=paramtest
